# Cytotoxicity and apoptogenic properties of the standardized extract of *Portulaca oleracea* on glioblastoma multiforme cancer cell line (U-87): a mechanistic study

**DOI:** 10.17179/excli2019-1063

**Published:** 2019-03-20

**Authors:** Vafa Baradaran Rahimi, Seyed Hadi Mousavi, Soroush Haghighi, Sina Soheili-Far, Vahid Reza Askari

**Affiliations:** 1 Pharmacological Research Center of Medicinal Plants, Mashhad University of Medical Sciences, Mashhad, Iran; 2 Student Research Committee, Department of Pharmacology, Faculty of Medicine, Mashhad University of Medical Sciences, Mashhad, Iran; 3 Medical Toxicology Research Center, Mashhad University of Medical Sciences, Mashhad, Iran; 4 Neurogenic Inflammation Research Center, Mashhad University of Medical Sciences, Mashhad, Iran

**Keywords:** glioblastoma multiforme (GBM), ROS, NF-kappaB, U-87 cell, Portulaca oleracea, nitric oxide

## Abstract

The traditional uses of *Portulaca oleracea L. *(PO) with anti-inflammatory and anti-cancer activity as well as antioxidants properties were expressed previously. Glioma is considered the most common primary brain tumor and its malignant form is the most lethal adult brain tumor, that glioblastoma covers about 50 % of glioma tumors. The present study was aimed to evaluate the cytotoxicity and apoptogenic effects of the hydro-ethanolic extract of PO on human glioblastoma cancer cell line (U-87) and the role of NF-κB. Cytotoxicity of the extract in the presence or absence of Vitamin C was evaluated using MTT assay, and the following hypotonic PI and SubG1 peak were performed. Moreover, the reactive oxygen species (ROS), the level of NF-κB protein and nitric oxide (NO) production were investigated. The extract had cytotoxicity and apoptogenic effects on U-87 cells in both the concentration and time-dependent manners. The mechanism of cytotoxicity and apoptosis induction of the extract at the first hours of incubation and low concentrations were dependent on ROS. However, the toxicity was replaced with NO pathway with time-lapse and higher concentrations. Results also indicated that the extract acts as an NF-κB inhibitor with concentration and time-dependent manners. The present study may suggest the anti-NF-κB activity of PO along with two upstream ROS and NO mechanisms. Furthermore, the extract as ethnobotanical may be used as adjunctive anti-cancer therapy against glioblastoma multiforme.

## Abbreviation List

Glioblastoma multiforme (GBM); Human umbilical vein endothelial cell (HUVEC); Inducible nitric oxide synthase (iNOS); Inhibitor of NF-κBα (IκBα); Intercellular adhesion molecule 1 (ICAM-1); Interleukin (IL); Monocyte chemoattractant protein-1 (MCP-1); Nitric oxide (NO); Nuclear factor-kappa B (NF-κB); *Portulaca oleracea L. *(*P. oleracea*, PO); Propidium iodide (PI); Reactive nitrite species (RNS); Reactive oxygen species (ROS); T helper (Th); Tumor necrosis factor-a (TNF-α); Vascular cell adhesion molecule 1 (VCAM-1) 

## Introduction

Glioma, the tumor of glial origin, is the most common type of primary brain tumors by 20 percent of total intracranial tumors (Nizamutdinov et al., 2017[41]). In particular, malignant glioma is considered as most causes of death type of adult brain tumors (Johung and Monje, 2017[31]; Yi et al., 2017[60]). More than 50 percent of all kinds of gliomas are dedicated to glioblastoma. Overall, the incidence of glioblastoma is 2-3 people in a hundred thousand and more common in men over sixty years old (Nizamutdinov et al., 2017[41]). Despite advances in understanding regarding the pathophysiology and therapy of glioblastoma, it is yet considered as an incurable disease, that unfortunately using the common medicines, removing the tumor using surgery and radiotherapy along with temozolomide (TMZ), the lifespan of patients will be not prolonged more than 12-15 months (Hegi and Stupp, 2015[29]). Therefore, further investigations are quite necessary to find new medications and methods for treating the disease. 

Reactive oxygen species (ROS) plays an important role in the pathogenesis of numerous human diseases including inflammatory states and particularly cancers. ROS expresses beneficial properties at the low level through adjusting intra-cellular signaling and homeostasis. Although, it could be carcinogenic regarding its damaging effects on proteins, lipids, and DNA at high concentrations (Acharya et al., 2010[2]). Various chemotherapies such as doxorubicin act through ROS over-production which causes cell death. ROS generation could be an appropriate pathway for cancer therapy, either by reducing antioxidants or ROS inducer agents (Prasad et al., 2017[44]). 

Nuclear factor-κB is a transcription factor which modifies many biological responses such as immune and inflammatory responses, cell growth and proliferation, differentiation and survival as well as apoptosis and cell adhesion (Nakajima and Kitamura, 2013[39]). It has been shown that aberrant NF-κB activation or its expression contributes to the development of different types of human tumors. Constitutively activated NF-κB transcription factor stimulates the expression of various genes being responsible for multiple aspects of tumor-genesis such as increased cancer cell proliferation, inhibition of apoptosis, enhancing tumor's angiogenesis and metastasis (Nelson et al., 2004[40]; Askari and Shafiee-Nick, 2019[12]). Thereby, NF-κB inhibitors are potential therapeutic candidates in cancer therapy through stopping tumor cell proliferation, tumor cell death and increasing sensitivity to anti-tumor agents (Escarcega et al., 2007[24]).

Medicinal plants have a long historical application in cancer treatment and natural sources are the origin of approximately 60 percent of anti-cancer drugs (Desai et al., 2008[23]).* Portulaca oleracea L *(PO) as folk medicine is an annual, succulent and wilding plant that belongs to Portulacaceae (Uddin et al., 2014[55]; Boskabady et al., 2016[18]; Hashemzehi et al., 2016[28]). The Iranian traditional medicine name of this plant is Qorfeh and also named Purslane in the English language. In ancient medical texts such as Qanoon e tib by Shaik Bu-Ali-Seena and Makhzan Ul Adviya by Mohammad Hussein Aghili Alavi Khorasani, it has been stated that PO could be used for fever, cramps, fumigation and burnt as well as inflammatory skin rash (Okwuasaba et al., 1987[42]; Rasheed et al., 2004[46]; Mohanapriya et al., 2006[38]; Baradaran Rahimi et al., 2019[16]). Moreover, PO is imported as a medicinal herb in the WHO due to its most benefits (Lim and Quah, 2007[36]). In previous studies, many pharmacological properties of PO have been demonstrated including anti-cancer, anti-inflammatory and strengthening the immune system (Yang et al., 2008[59], Zhu and Wu, 2009[66], Baradaran Rahimi et al., 2019[16]). Also, PO blocked the HepG2 and HeLa cells proliferation (Chen et al., 2010[21]). Recently, it has been emphasized that ethanolic extract of PO possess anti-inflammatory and antiproliferative effects on human lymphocytes as well as regulating the Th1/Th2 balance toward Th1 (Askari et al., 2016[10]). Flavonoids are one of the most abundant and important active constituents of PO. Kaempferol and apigenin have been mainly isolated from leaf and stem (Zhu et al., 2010[65]). Also, Luteolin, myricetin, quercetin, genistein, and genistin (Zhu et al., 2010[65]) have been derived from the whole plant. Portulacanones A, portulacanones B, portulacanones C, portulacanones D, and 2,2'-Dihydroxy-4',6'-dimethoxychalcone have been isolated from aerial parts of PO (Yan et al., 2012[58]).

Altogether, it is necessary to investigate new treatments regarding the incurability of the tumor. The present study was conducted to investigate the cytotoxicity and apoptogenic properties of PO and also the role of NF-κB on glioblastoma multiform (U-87) cell line.

## Material and Methods

### Drugs and chemicals

Roswell Park Memorial Institute medium (RPMI, code 61-870-036) and fetal bovine serum (FBS, code 10270-106) were bought from Gibco Life Technologies (Grand Island, NY, USA). 3- (4, 5-dimethylthiazol-2-yl)-2, 5-diphenyltetrazolium bromide (MTT, code M-5655), Dimethyl sulfoxide (DMSO, code D4540), penicillin-streptomycin (code P4333) and 2′, 7′-Dichlorofluorescin diacetate (DCFH-DA, code D6883) were purchased from Sigma-Aldrich (St. Louis, MO). Ethanol (code 1009832500) was obtained from Merck (Darmstadt, Germany).

### Preparation of plant extract

**Figure 1:**
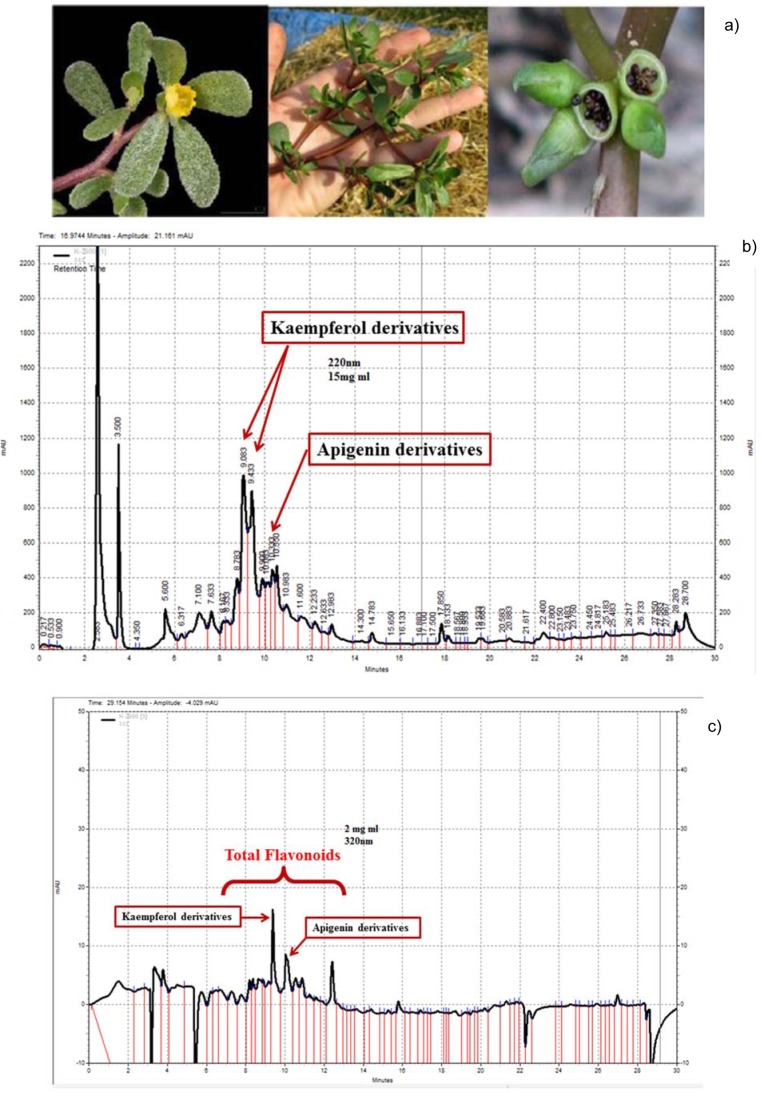
a) Different parts of PO (Askari et al., 2016a). b) HPLC fingerprints of PO at 220 nm. c) HPLC fingerprints of PO at 320 nm

PO (Figure 1(Fig. 1); Reference in Figure 1: Askari et al., 2016[10]) was collected from Sabzevar, Khorasan Razavi, Iran in the month of July 2016 (herbarium No. 12-1615-240). The plant was identified by Mr. Jouharchi, and a voucher of a sample was served as references in the herbarium of the school of pharmacy at Mashhad University of Medical Sciences (Deposition/Herbarium No: 12-1615-240). The leaves were dried in shadow and powdered and extraction was performed by the maceration method (Askari et al. 2016[7]; Boskabady et al., 2016[18], Baradaran Rahimi et al., 2019[16]). 100 g of leaves powder was soaked with 1 lit 70 % ethanol for 48 h at controlled room temperature. The extract was concentrated via rotary evaporator and then freeze-dried. The yield of dried extract was 17.5 % w/w.

### Measurement of Total Phenolic Content (TPC) of PO

Using Folin-Ciocalteu (FC) reagent, the TPC was determined according to the previous studies with minor modification (Ainsworth and Gillespie, 2007[3]; Rover and Brown, 2013[48]). In brief, one hundred microliters of the extract (20 µg/mL) were combined with the same volume of water in a test tube. Then, about 200 µL of FC reagent was added to the tube with following 2600 µL of 5 % (w/v) sodium carbonate solution. The mixture was incubated at 40 ºC for 20 min along with fine shaking. The tubes were then quickly cooled and the developed color was read at 760 nm using a MultiSpec UV-Vis spectrophotometer (Shimadzu, Tokyo, Japan). Estimation of phenolic compounds was carried out regarding polyphenol reference calibration curve of the ethanolic solution of Gallic acid (GA) in a range of 0.5 to 10 mg/L (Rover and Brown, 2013[48]; Abbasi et al., 2017[1]; Baradaran Rahimi et al., 2019[16]). The amount of TPC was measured regarding mg of GA equivalent (GAE) per gram of dry extract. An identical process was performed for blank using of 100 µl of distilled water instead of the extract. 

### HPLC finger print of PO

The liquid chromatography including of the model 510 waters pump (Waters Association, Milford, MA/USA), a variable wavelength, the model 486 waters UV detector and the U6K, waters sample injection system. The mobile phase was a mixture of methanol: acetonitrile: tetrahydrofuran: 0.5 % glacial acetic acid (5:3:18:74). The mobile phase was filtered under vacuum, degassed and pumped through the Novapak C18 column (150×3.9 mm i.d.) at a flow rate of 1.0 ml/min. The chromatograms were recorded at 220 and 320 nm (Zhao et al., 2013[62]; Askari et al., 2016[10]; Baradaran Rahimi et al., 2019[16]). HPLC fingerprints and partial characterization were performed with the cooperation of Gloexir Pars ® Company (220 and 320 nm, Figures 1B and C(Fig. 1)).

### Cell culture

U-87 human primary glioblastoma cell line was obtained from Pasteur Institute, Tehran, Iran. U-87 cells were cultured in RPMI-1640 medium (10 % FBS, 100 U/ml penicillin, 100 µg/ml streptomycin, 2 mM L-glutamine, and 1 mM sodium pyruvate and HEPES buffer) at 37 °C in 5 % v/v CO_2_.

### Cell viability assay

Cytotoxicity evaluation was examined using MTT assay as described previously (Askari et al., 2016[7]). Approximately 8 × 10^3^ cells were seeded in 96 well plates and incubated with PO extract series of dilution (0.78 to 800 μg/mL) and doxorubicin (0.25 and 0.5 μg/mL) for 24, 48 and 72 h at 37 °C in 5 % CO_2_ incubator. After that, 10 μL of MTT reagent (5 mg/ml) was added to each well and was further incubated for 1 h. Formazan crystals were dissolved in 100 μL DMSO and the absorbance was read using StatFAX 2100 ELISA plate reader (Awareness Inc., USA) at 570 nm in referencing 620 nm. Half-maximal inhibitory concentration (IC_50_) for each time exposer (24, 48 and 72 h) was assessed using GraphPad Prism® 6.01 (GraphPad Software, San Diego, CA) software.

### Propidium iodide staining 

The apoptotic cells were detected using propidium iodide (PI) staining of treated cells, followed by flow cytometry to evaluate the so-called sub-G1 peak (Vazifedan et al., 2017[56]). Briefly, U-87 cells (10^5^ cells/well) were cultured in 24 well plates for 24 h. Next, cells were treated with different concentrations of PO and incubated for 48 and 72 h. Then cells were washed with PBS and were incubated with 400 μL of a hypotonic buffer (50 μg/mL PI in 0.1 % sodium citrate and 0.1 % Triton X-100) at 4 °C overnight in the dark and were analyzed using FACScan flow cytometer. Considering the cell proliferation results and IC_50_ for PO, the concentrations lesser, equal and more than IC_50_ in a specific ratio were evaluated for Sub-G1 peak and percentage of apoptosis. Flow-cytometry histograms were analyzed using WinMDI Version 2.8 and the percentages of apoptosis cells were determined.

### NF-κB nuclear extraction and assessment of its amount

The Nuclear Extraction Kit (catalog no. ab113474) and NF-κB Transcription Factor Assay kit (catalog no. ab133112) were purchased from Abcam (Cambridge, MA, USA). Bay11-7082 (25 µM) was used as an NF-κB inhibitor. The levels of NF-κB in the cytosol and nuclear were measured by ELISA according to the manufacturer's protocol. In addition, to evaluate the effect of PO extract on either nuclear localization or expression of NF-κB, the nuclear/cytosolic ratio was assessed. 

### Measurement of Reactive Oxygen Species (ROS) level

Intra-cellular ROS level was determined using DCFH-DA as described previously (Rahimi et al., 2017[45]; Askari and Shafiee-Nick, 2019[11][12]). 10^4 ^U-87 cells were cultured in 96 well-plates. After 24 h, the cells were treated with different concentrations of PO extract and vitamin C (10 µM) as a positive control for 24, 48 and 72 h. After that, DCFH-DA was added to cells and incubated for 30 min. The intensities were read using ELISA reader at excitation wavelength of 504 nm and the emission wavelength of 524 nm. 

### Assessment of nitric oxide level

The level of nitric oxide was assessed using the Griess reaction which indirectly measured nitric oxide level through the accumulation of sodium nitrite. Sodium nitrite is a stable final product in the cell culture (Arancibia et al., 2016[5]). U-87 cells were seeded in 96 well plates and treated with different PO extract concentrations for 24, 48 and 72 h. Next, the supernatants were isolated and mixed with the Griess reagent at room temperature for 5 min and the absorbance was read with ELISA reader (Sharifi et al., 2004[49]; Askari and Shafiee-Nick, 2019[11]). 

### Human lymphocytes isolation culturing and cytotoxicity assessment 

Fifteen ml of brachial vein whole blood were prepared from each subject and collected into a heparinized tube. Peripheral blood mononuclear cells (PBMC) were separated using Ficoll density gradient as described previously (Boskabady et al., 2011[19]; Guo et al., 2015[26]; Askari et al.; 2018[8][9]). The live cells were counted using trypan blue solution % 0.04. The cell viability and the cell numbers were more than 98 % and 2.5×10^6^ cells/mL, respectively in all samples (Guo et al., 2015[26], Askari et al., 2016[10]). The proliferation of isolated lymphocytes was assessed using colorimetric WST-1 assay kit (Roche Diagnostic, Mannheim, Germany) according to manufacturer's instruction. Briefly, cells were triply cultured at 10^5^ cell/100 µl per each 96-well culture plate and treated with different concentrations of PO extract (0-800 µg/ml) in the final volume of 200 µl per well and incubated for 24 and 48 h. After that, cells were incubated with 10 % v/v of the final volume of each well with WST-1 reagent for 4 hrs. Finally, the optical density was measured at 450 nm using reference at 630 nm (Askari et al., 2018[8][9]).

### Statistical analysis

Data analyses were done using GraphPad Prism ® 6 (GraphPad Software, San Diego, CA) software and results were expressed as means ± SEM. Firstly, the normality of distribution was assessed using the Kolmogorov-Smirnov test. After passing the test and possessing normal distribution, group-data in comparison to untreated (control) group were performed using one-way analysis of variance (ANOVA) with Dunnett's *post hoc* test. P<0.05 was considered significant (Askari and Shafiee-Nick, 2019[12]; Baradaran Rahimi et al., 2018[15]).

### Ethical disclosures 

The study was executed in accordance with ethical guidelines approved by the Ethical Committee of Mashhad University of Medical Sciences (IR.MUMS.fm.REC.1395.340) on 7 December 2016.

## Results

### HPLC fingerprints and total phenolic contents of PO extract

Using the maceration method, the yield of extract was 17.5 % in the proportion of raw dried PO. TPC was also determined 9.1 ± 0.45 as mg GAE/g dried extract (Table 1(Tab. 1)). A simple and also reliable HPLC fingerprint has been performed for qualification and total flavonoid content at 220 and 320 nm, respectively. Moreover, regarding the standard of kaempferol and apigenin and our previous study (Askari et al., 2016[10]), the pikes at 9.083 and 9.433 min, and 10.083 and 10.333 min were characterized to kaempferol and apigenin derivatives.

### PO significantly mitigated U-87 cell proliferation after 24, 48 and 72 hours incubation

**Figure 2:**
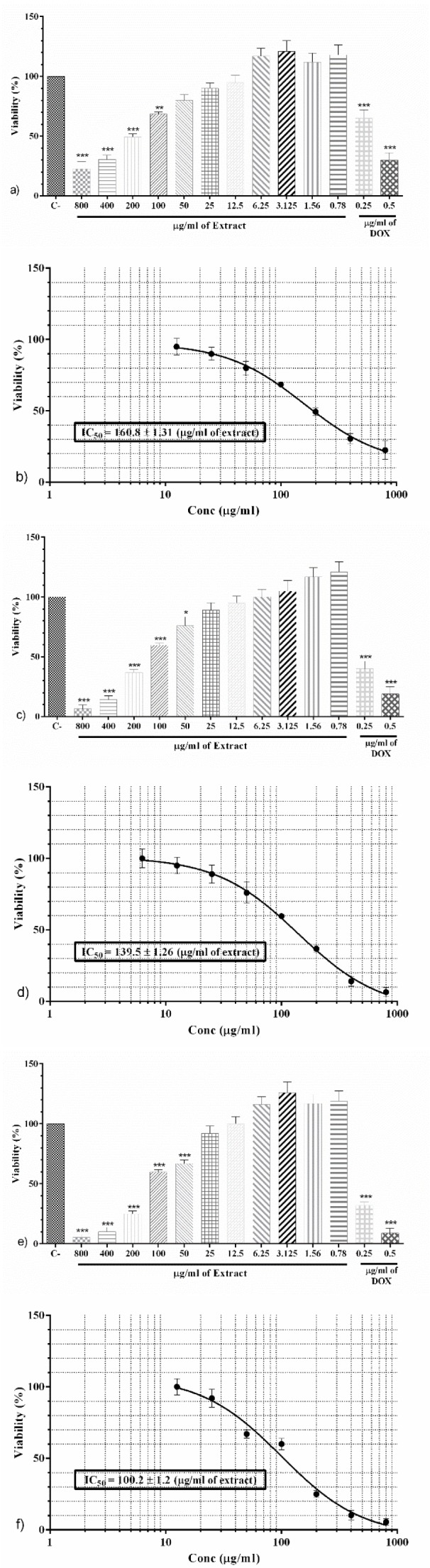
a) The effect of PO and doxorubicin on cell proliferation after 24 h. b) assessment of IC_50_ of PO on cell proliferation after 24 h. c) The effect of PO and doxorubicin on cell proliferation after 48 h. d) assessment of IC_50_ of PO on cell proliferation after 48 h. e) Effect of PO and doxorubicin on cell proliferation after 72 h. f) assessment of IC_50_ of PO on cell proliferation after 72 h. Data were presented as mean ± SEM. P<0.05 *, p<0.01 ** and p<0.001 *** as compared with the control group. (n=5)

**After 24 h; **PO extract (100-800 μg/ml) significantly diminished U-87 cell proliferation in a concentration-dependent manner (p<0.01 to p<0.001, Figure 2a(Fig. 2)). Besides, doxorubicin (0.25 and 0.5 μg/ml) as positive control significantly reduced cell proliferation (p<0.001, Figure 2a(Fig. 2)). IC_50_ for 24 h incubation was considered 160.8 ± 1.31 μg/ml using Prism (GraphPad, Version 6. 00, Figure 2b(Fig. 2)). 

**After 48 h; **As shown in Figure 2c(Fig. 2), PO extract (50-800 μg/ml) significantly decreased U-87 cell proliferation in a concentration-dependent manner (p<0.05 to p<0.001). Furthermore, doxorubicin (0.25 and 0.5 μg/ml) as positive control significantly reduced cell proliferation (p<0.001, Figure 2c(Fig. 2)). IC_50_ for 48 h incubation was measured 139.5 ± 1.26 μg/ml using Prism (GraphPad, Version 6. 00, Figure 2d(Fig. 2)). 

**After 72 h; **As described in Figure 2e(Fig. 2), PO extract (50-800 μg/ml) as well as doxorubicin (0.25 and 0.5 μg/ml) significantly and dose-dependently diminished U-87 cell proliferation (p<0.001). Using Prism (GraphPad, Version 6. 00), IC_50_ for 72 h incubation was considered 100.2 ± 1.2 μg/ml (Figure 2f(Fig. 2)).

### PO markedly augmented U-87 apoptosis through PI staining after 48 and 72 hours incubation

**Figure 3:**
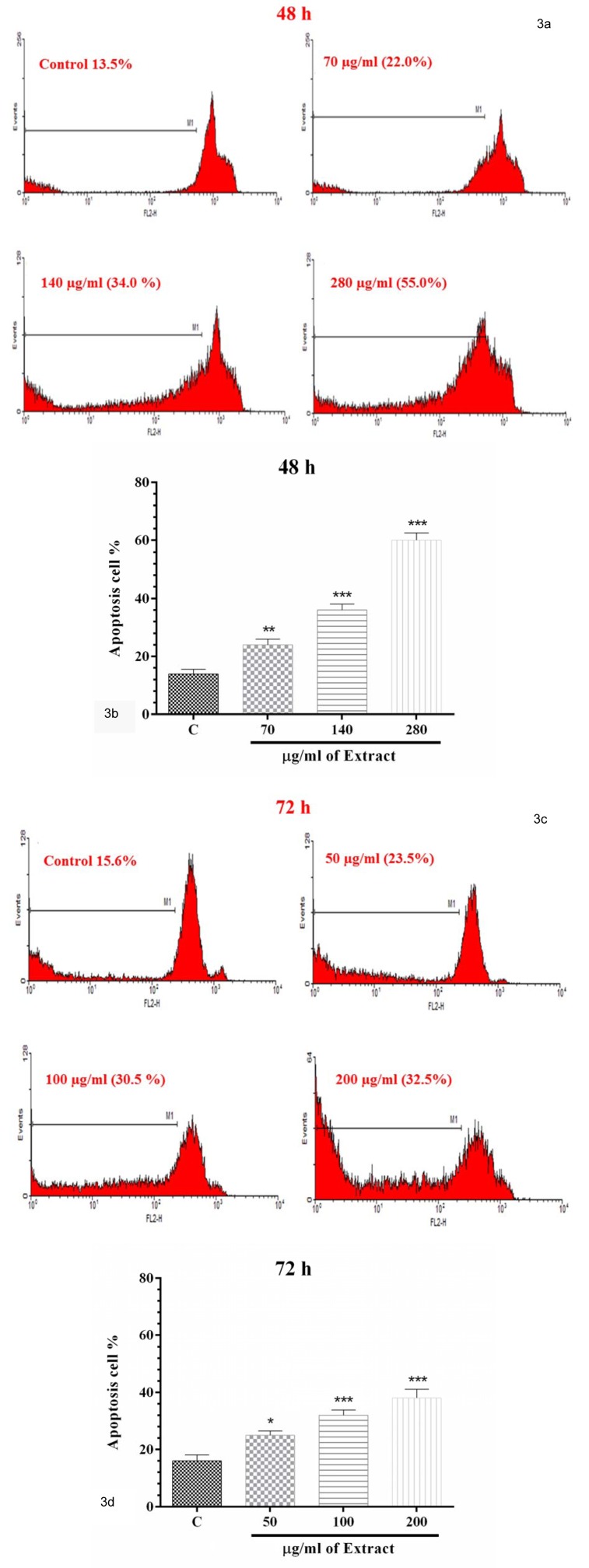
a) Flow cytometry histogram for negative control, concentrations 70, 140 and 280 μg/ml of PO after 48 h. b) The percentage of apoptotic cells after 48 h incubation with negative control, concentrations 70, 140 and 280 μg/ml of PO after 48 h. c) Flow cytometry histogram for negative control, concentrations 50, 100 and 200 μg/ml of PO after 72 h. d) The percentage of apoptotic cells after 72 h incubation with negative control, concentrations 50, 100 and 200 μg/ml of PO. Data were presented as mean ± SEM. P<0.05 *, p<0.01 ** and p<0.001 *** as compared with control group. (n=3)

**After 48 h;** In comparison with control group, all three concentrations of PO (70, 140 and 280 μg/ml) markedly enhanced the percentages of apoptotic cells (p<0.01 to p<0.001, respectively, Figures 3a and b(Fig. 3)).

**After 72 h; **All three concentrations of PO (50, 100 and 200 μg/ml) significantly raised the percentages of apoptotic cells in comparison to control group (p<0.05 to p<0.001, respectively, Figures 3c and d(Fig. 3)).

### Effects of PO and vitamin C on ROS level and cell proliferation after 24, 48 and 72 hours incubation 

**Figure 4:**
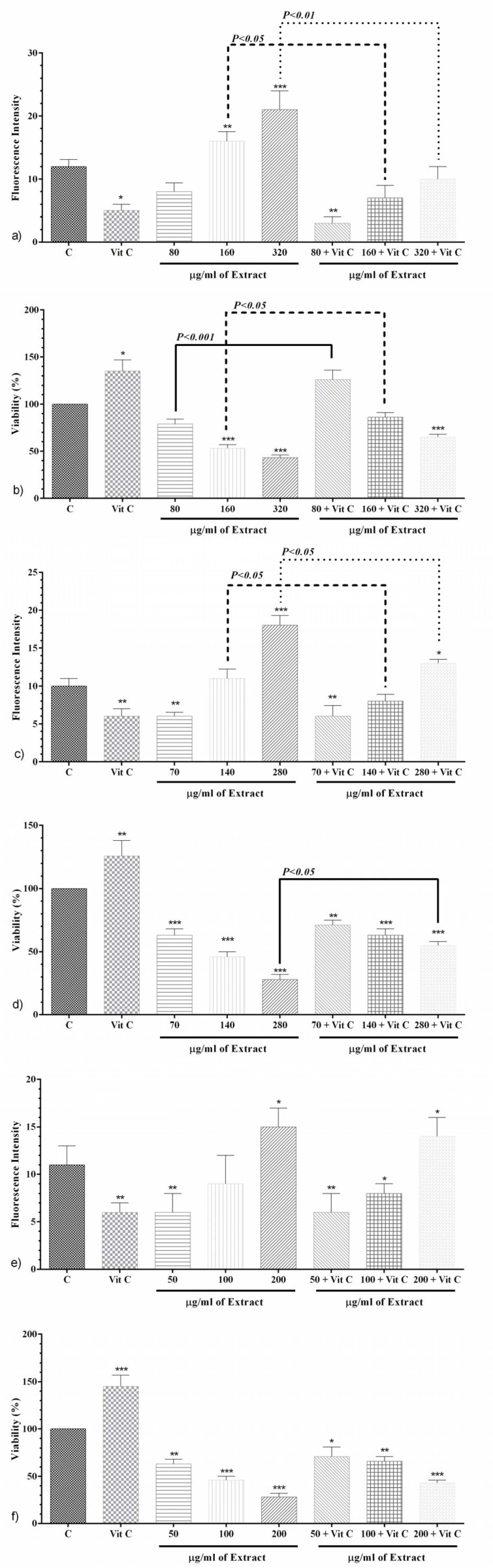
The effects of PO and vitamin C on ROS level (a) and cell proliferation (b) after 24 h; The effects of PO and vitamin C on ROS level (c) and cell proliferation (d) after 48 h; The effects of PO and vitamin C on ROS level (e) and cell proliferation (f) after 72 h. Data were presented as mean ± SEM. P<0.05 *, p<0.01 ** and p<0.001 *** as compared with the control group. (n=5)

**After 24 h;** Vitamin C (10 μM) made a significant decrement in ROS level (p<0.05, Figure 4a(Fig. 4)), whereas it made a significant increment in cell proliferation (p<0.05, Figure 4b(Fig. 4)). Co-treatment of PO (80 μg/ml) with vitamin C (10μM) significantly mitigated ROS level compared to negative control group (p<0.01, Figure 4a(Fig. 4)).

Furthermore, co-treatment of PO (80 μg/ml) with vitamin C (10 μM) markedly improved cell proliferation in comparison with PO (80 μg/ml) without vitamin C group (p<0.001, Figure 4b(Fig. 4)). In contrast, PO (160 and 320 μg/ml) significantly increased ROS level and decreased cell proliferation in comparison with negative control group (p<0.01 to p<0.001, respectively, Figures 4a and b(Fig. 4)). Co-treatment of PO (160 and 320 μg/ml) with vitamin C (10 μM) markedly diminished ROS level compared to PO alone (p<0.05 and p<0.01, respectively, Figure 4a(Fig. 4)). Also, cell proliferation was significantly alleviated in co-treatment of PO (160 μg/ml) with vitamin C (p<0.05, Figure 4b(Fig. 4)). 

**After 48 h; **Vitamin C made a significant decrement in ROS level (p<0.01, Figure 4c(Fig. 4)), while it made a significant increment in cell proliferation (p<0.01, Figure 4d(Fig. 4)). PO (70 μg/ml) significantly attenuated ROS level (Figure 4c(Fig. 4)) and cell proliferation (Figure 4d(Fig. 4)) in comparison with control group (p<0.01 and p<0.001, respectively). Also, co-treatment of PO (70 μg/ml) with vitamin C (10 μM) markedly reduced ROS level (Figure 4c(Fig. 4)) and cell proliferation (Figure 4d(Fig. 4)) compared to control group (p<0.01 for both cases). PO (140 μg/ml) as well as PO (140 μg/ml) with vitamin C (10μM) had no significant effects on ROS level (Figure 4c(Fig. 4)), whereas markedly mitigated cell proliferation (p<0.001, Figure 4d(Fig. 4)). PO (280 μg/ml) significantly raised ROS level (p<0.001) compared to control group, which is markedly decreased in the presence of vitamin C (p<0.05, Figure 4c(Fig. 4)). Moreover, cell proliferation markedly alleviated with PO (280 μg/ml, p<0.001) compared to the control group, which is significantly appended in the presence of vitamin C (p<0.001, Figure 4c(Fig. 4)). 

**After 72 h; **Vitamin C (10 μM) after 72 h made a significant decrement in ROS level (p<0.01, Figure 4e(Fig. 4)), whereas it made a significant increment in cell proliferation (p<0.001, Figure 4f(Fig. 4)). PO (50 μg/ml) singly and along with vitamin C notably reduced ROS level (p<0.01 for both cases, Figure 4e(Fig. 4)). PO (100 μg/ml) had no significant effect on ROS level, however co-treatment with vitamin C attenuated the effect of PO (p<0.05, Figure 4e(Fig. 4)). Either PO (200 μg/ml) alone or in combination to vitamin C notably reduced ROS level compared to the control group (p<0.05 for both cases, Figure 4e(Fig. 4)). In comparison to the control group, PO (50, 100 and 200 μg/ml) alone and accompanying vitamin C markedly alleviated cell proliferation (p<0.05 to p<0.001, Figure 4f(Fig. 4)).

### PO significantly attenuated NF-κB expression but not nuclear/cytosol ratio

**Figure 5:**
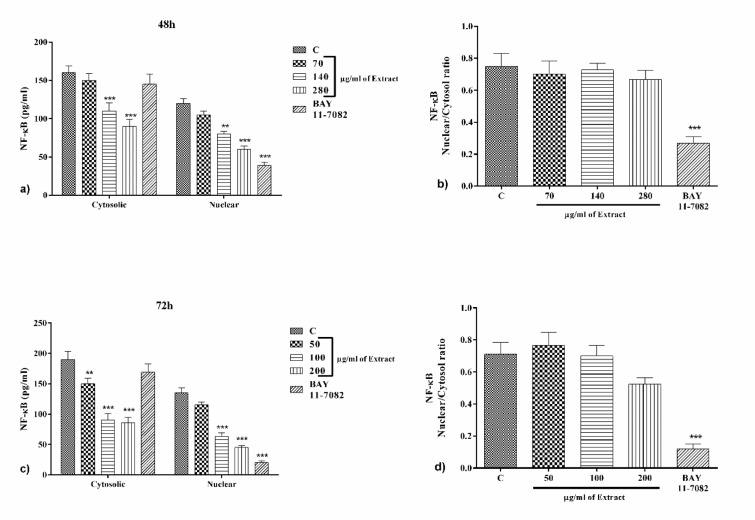
The effect of PO on the levels of NF-κB in the cytosol and nuclear after 48 h (a) and 72 h (c) incubation with different concentrations of PO and BAY 11-7082, as well as nuclear/cytosol ratio of NF-κB after 48 h (b) and 72 h (d). Data were presented as mean ± SEM. P<0.01 ** and p<0.001 *** as compared with the control group. (n=6)

Bay11-7082 (25 μM) significantly alleviated the level of nuclear NF-κB and nuclear/cytosol ratio compared to the control group after 48 and 72 h (p<0.001, Figure 5a-d(Fig. 5)). Incubation with PO extract (140 and 280 µg/ml, and 50-200 µg/ml for 48 h and 72 h incubation, respectively) significantly reduced nuclear and cytosolic levels of NF-κb comparing to control group (p<0.001 to 0.01, for all cases, Figure 5a and c(Fig. 5)). Notably, the nuclear/cytosol ratio of NF-κb was not considered significant for all tested concentrations of the extract when compared to the control group (Figure 5a and d(Fig. 5)).

### PO markedly appended NO production

**Figure 6:**
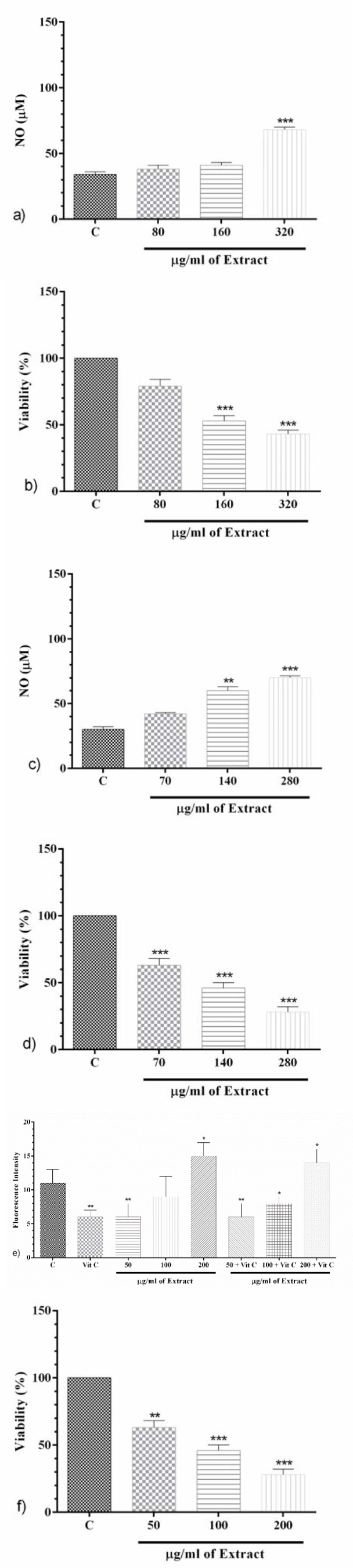
The effect of PO on NO production (a) and cell proliferation (b) after 24 h; the effect of PO on NO production (c) and cell proliferation (d) after 48 h; the effect of PO on NO production (e) and cell proliferation (f) after 72 h. Data were presented as mean ± SEM. P<0.01 ** and p<0.001 *** as compared with the control group. (n=5)

**After 24 h; **PO (320 μg/ml) significantly improved NO level compared to the control group (p<0.001, Figure 6a(Fig. 6)). Furthermore, PO (160 and 320 μg/ml) made a significant decrement on the level of cell proliferation (p<0.001 for both, Figure 6b(Fig. 6)).

**After 48 h; **PO (140 and 280 μg/ml) significantly raised NO level (p<0.01 and p<0.001, respectively, Figure 6c(Fig. 6)) compared to the control group. Also, PO (70, 140 and 280 μg/ml) significantly decreased cell proliferation compared to the control group (p<0.001 for all, Figure 6d(Fig. 6)).

**After 72 h; **PO (50, 100 and 200 μg/ml) markedly increased NO level compared to the control group (p<0.001, Figure 6e(Fig. 6)), and made a significant decrement in cell proliferation (p<0.01 to p<0.001, Figure 6f(Fig. 6)).

### Effect of PO on lymphocytes' cell viability

**Figure 7:**
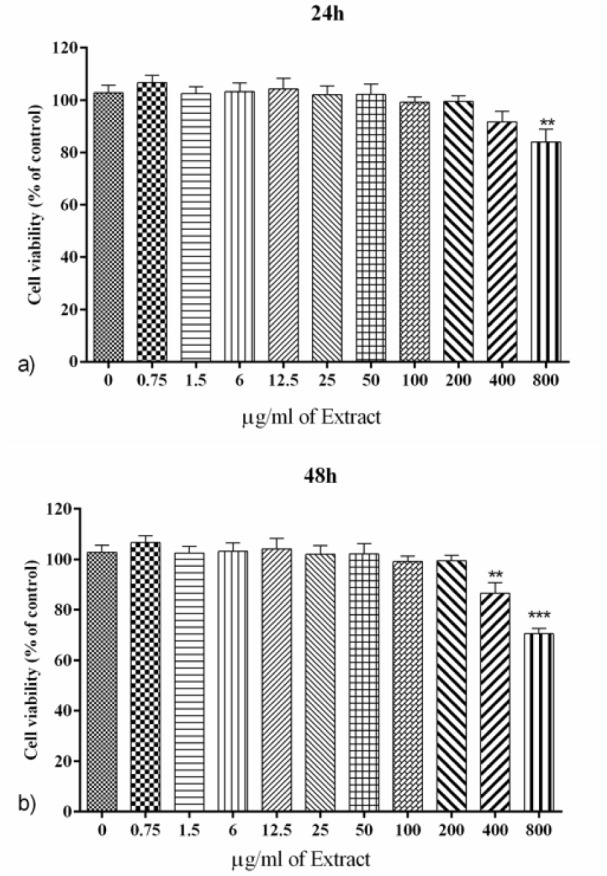
The effect of PO extract on lymphocytes viability after a) 24 h. and b) 48 h. Data were presented as mean ± SEM. P<0.01 ** and p<0.001 *** as compared with the control group. (n=6)

The cell viability of lymphocytes was assessed after 24 and 48 h incubation with PO extract (0-800 µg/ml). Our records indicated that incubation with PO extract (≤400 and ≤200 µg/ml for 24 and 48 h, respectively) had no cytotoxicity effect on isolated human lymphocytes, although the concentrations of 400 and 800 µg/ml significantly reduced the cell viability after 48 h incubation (p<0.001 to 0.01 for both cases, Figures 7a and b(Fig. 7)).

## Discussion

To our knowledge, this is the first study evaluating the cytotoxic effects of PO on human glioblastoma (U-87) cell line. In the present study, we determined the cytotoxic effects of the hydroethanolic extract of PO using the MTT method. To achieve better insight into the cytotoxicity mechanisms, apoptosis using hypotonic PI and SubG1 peak and ROS production as well as levels of nuclear and cytosolic of NF-κB were examined. Moreover, we found that PO extract at toxic concentrations for glioblastoma cells possess no cytotoxicity for isolated human lymphocytes as normal cells. 

U-87 is one of the most common cell lines used in glioblastoma studies which is derived from a female pleomorphic glioma patient. Our results indicated that PO significantly reduced cell proliferation after 24, 48 and 72 h incubation with a concentration and time-dependent manner. Using Prism (GraphPad, Version 6. 00), the levels of IC_50_ for 24, 48 and 72 h incubation were approximately estimated 161, 140 and 100 μg/ml, respectively. In addition, doxorubicin at concentrations of 0.25 and 0.5 μg/ml, as a positive control drug with proven cytotoxic effects, was markedly diminished cell proliferation. Askari et al. (2016[10]) showed that hydro-ethanolic extract of PO significantly decreased mitogen-activated T lymphocytes proliferation as a model of cancer. Moreover, Chen and coworkers suggested that PO inhibits progression and cell proliferation in rats with ovarian cancer in a concentration-dependent manner (Chen et al., 2010[21]). Furthermore, it has been demonstrated that polysaccharides contained in PO encompass various properties including anti-cancer, anti-inflammatory and increased immunity (Yang et al., 2008[59]; Zhu and Wu, 2009[66]). In another study, PO seed oil leads to cytotoxicity in human liver cancer (HepG2) and human lung cancer (A-549) cells (Al-Sheddi et al., 2015[4]). 

Given the nature of apoptosis depending on time and concentration, IC_50_ has assumed for each time exposed. As mentioned in the result section, IC_50_ values were reduced depending on the passage of time. Based on pieces of evidence, hypotonic PI is conducted for evaluating mechanistic effects of cytotoxicity, which represents late apoptosis (Bakhtiari et al., 2016[13]). Our results suggested that apoptosis was inducted in U-87 cells after 48 and 72 h in a concentration and time-dependent manner. It is concluded that apoptosis-inducing concentrations being less by the passage of time. Interestingly, we found that the percentage of the apoptotic cell was higher in 48 h incubation rather than 72 h, however, the cytotoxicity was higher in 72 h incubation rather than 48 h. It means that apoptosis is not the sole cause of cell death. In line with our findings, Zhao et al. showed that polysaccharides derived from PO induce apoptosis in cervical carcinoma U14 and HeLa cells through decreasing Bcl-2, as an anti-apoptotic protein, and increasing Bax, as an apoptotic protein, as well as increasing Bax/Bcl-2 ratio (Zhao et al., 2013[62], 2017[63]). Zheng et al. (2014[64]) showed that Portulacerebroside A (PCA), a new cerebroside compound isolated from PO, markedly diminished human liver cancer HCCLM3 cell viability in time and concentration-dependent manner. PCA raises the percentage of apoptotic cells, disrupted mitochondrial membrane permeability and stimulates the release of mitochondrial cytochrome C and AIF as well as increased caspase-9 and caspase-3. It is concluded that PCA is a promising candidate for treating liver cancer (Zheng et al., 2014[64]). In agreement to our characterizing the PO extract, it has been shown that aerial parts of PO extract include flavonoids such as apigenin, kaempferol and their polyphenolic derivatives (Askari et al., 2016[10]; Boskabady et al., 2016[18]; Hashemzehi et al., 2016[28]). Recently, it has been shown that apigenin potently and significantly reduces cell proliferation of human glioblastoma cell lines through inducing the apoptosis and inhibition of AKT/mTOR signaling pathways (Coelho et al., 2016[22]; Kim et al., 2016[33]; Stump et al., 2017[54]). Furthermore, kaempferol has indicated anti-glioma activity through induction of apoptosis and ROS generation (Sharma et al., 2007[50]). Thereby, polyphenolic compounds such as kaempferol and apigenin, and polysaccharides accompany cytotoxicity effects of PO on human glioma cells.

The level of ROS in tumor cells is usually higher than normal cells; accordingly, they are more sensitive to oxidative stress mediated by anti-tumor drugs and apoptosis in these cells rises by ROS (Hosseini et al., 2017[30]). It has been demonstrated that herbal compounds such as phenols induce apoptosis generated by ROS (Garg et al., 2005[25]). ROS over-production leads to increased release of cytochrome C, increased activity of caspase proteins and cell death (Cadenas, 2004[20]). In this study, intracellular ROS production was measured to explore the role of ROS in apoptosis. ROS level was evaluated in different concentrations of PO, and vitamin C (10 μM) as positive control after 24, 48 and 72 h, using DCFH-DA fluorescent dye along with cell proliferation using MTT method. Interestingly, PO (160 and 320 μg/ml) significantly improved ROS production after 24 h, which is reversed by vitamin C as an anti-oxidant. It means that ROS may play an important role to initiate the cytotoxicity and apoptosis. Regarding prove the mechanism of cytotoxicity, viability was measured using the same concentrations in the presence or absence of vitamin C as a radical scavenger. The results showed that two higher concentrations (160 and 320 µg/ml) of PO reduced cell viability being reversed by vitamin C. Although, the abolishment of cytotoxicity by vitamin C for 320 µg/ml of PO is not statistically significant, this may indicate that ROS is not the sole mechanism for cell death. After 48 h, the highest concentration of PO (280 μg/ml) markedly raised ROS production, which is accompanied by a decrement in ROS level and increment in cell proliferation after vitamin C addition. It can be deduced that presumably after 48 h, PO leads to cell death through other pathways, except ROS production. After 72 h, only the highest concentration of PO (200 μg/ml) notably propagated ROS level which had not been reduced by vitamin C addition. Our results revealed that after 72 h, vitamin C could not make a significant change in cell proliferation as an anti-oxidant and ROS reducer.

It has been demonstrated that ROS signaling is concentration-dependent. In low concentrations, oxidative stress and ROS leads to increased cell survival through appended activation of protein kinase D followed by NF-κB activation. Activated NF-κB enhanced anti-oxidative proteins such as magnesium superoxide dismutase (MnSOD) and anti-apoptotic proteins including A20 and cIAPs (Storz and Toker, 2003[53]; Storz et al., 2004[51][52]). NF-κB plays a dual role in cancer. NF-κB is a part of the defense and immune system which removes abnormal cells. On the other hand, NF-κB is activated at numerous cancers and has a wide spectrum of carcinogenic properties. NF-κB transcription factor is capable of making all necessary changes for cancerous cells (Karin et al., 2002[32]). Growth and cell division are stimulated by NF-κB through enabling IL-2, GM-CSF and CD40L genes. NF-κB activation is related to cancers including breast, prostate, pancreas, colorectal and glioblastoma (Park and Hong, 2016[43]). Cancers with activated NF-κB usually exhibit resistance to chemotherapy likely via p-glycoprotein. Inhibitors of NF-κB have special influence in cancer treatment (Bakhtiari et al., 2015[14], 2016[13]). Chemotherapy drugs lead to ROS and nitric oxide over-production which could mitigate NF-κB through TRAF4 (TNF receptor-associated factor 4) (Liou and Storz, 2010[37]).

In the present study, the effects of PO on levels of nuclear and cytosolic of NF-κB as well as nuclear/cytosolic ratio were evaluated after 48 and 72 h incubation. The results indicated that PO extract at 140 and 280 μg/ml after 48 h, and all three concentrations of PO (50, 100 and 200 μg/ml) after 72 h significantly diminished both of nuclear and cytosolic levels of NF-κB, although did not alter the nuclear/cytosolic ratio of NF-κB. It shows that PO affects the expression of NF-κB rather than nuclear translocation. In concordance to our study, the effects of PO have been shown in many studies. Zhao et al. (2017[63]) suggested that polysaccharides proportion of PO attenuates NF-κB expression and also provides cell death in HeLa cell line. Also, α-linolenic acid, as one of the compounds found abundantly in PO, could alleviate COX-2 and NF-κB gene expression and MAP kinase pathways (Ren and Chung, 2007[47]). It has been demonstrated that PO made a decrement in T lymphocytes proliferation, an increment in Th1/Th2 ratio toward to Th1 and anti-cancer effects (Askari et al., 2016[10]). Moreover, Guoyin et al. (2017[27]) showed that PO decreased interleukin-6 (IL-6), IL-1β, tumor necrosis factor-α (TNF-α) in N-nitrosodiethylamine (NDEA) induced hepatocellular carcinomas (HCC). Furthermore, PO markedly reduced MDA and raised SOD in serum. PO markedly diminished NF-κB and inhibitor of NF-κBα (IκBα) expression and enhanced Nrf2 expression. It is concluded that PO possesses anti-oxidant and anti-inflammatory properties in hepatocellular carcinomas (Guoyin et al., 2017[27]). It has been demonstrated that PO polysaccharides have a strong scavenging activity against superoxide anion, nitric oxide, hydroxyl and 1, 1-diphenyl-2-picrylhydrazyl radicals. Furthermore, PO polysaccharides enhanced blood B and T lymphocytes and thymocytes isolated from rats with ovarian cancer proliferation as well as mitigated red blood cell hemolysis. It is revealed that PO polysaccharides could be a therapeutic significance for ovarian cancer therapy through strong free radical scavenging and improving immunity functions (YouGuo et al., 2009[61]). In addition, kaempferol (Kim et al., 2010[34]; Basu et al., 2017[17]) and apigenin (Wang et al., 2014[57]) derivatives which were found in PO extract have shown inhibitory effects on both the expression and nuclear translocation of NF-κB in various experiments. 

Due to completing the mechanisms accountable for PO anti-cancer activity, we measured the effects of PO on nitric oxide and related toxicity. We found that PO concentration and time-dependently increased the level of nitric oxide metabolites using Griess reaction. Increasingly, we observed that initial cytotoxicity and apoptogenic activity of PO at first onset are related to ROS production, while during the time, production of NO significantly raised and may be responsible for its cytotoxicity. The study showed that co-addition of vitamin C and PO on glioblastoma cells could not compensate for the reduction of cell viability at higher concentrations and also a long time. Furthermore, the lower concentrations of PO extract which did not augment ROS production decreased cell viability and enhanced NO metabolites concentration. Likely, the subsequent mechanism of ROS is reactive nitrite species (RNS). However, further studies are needed to exactly prove the cytotoxicity including an antagonist of NO synthesis (NOS, e.g. L-NAME) and expression of inducible NOS (iNOS) using Western blotting or ELISA methods. It has been demonstrated that U-87 cells possess a high capacity for migration and invasion (Li et al., 2017[35]; Arif et al., 2017[6]). However, as another limitation of our study, migration, and invasiveness of this cell in presence of PO extract should be examined in future studies. After the literature review, we have shown that there are no studies on the use of PO on glioblastoma multiforme cancer cell line. Another noteworthy limitation of our study is that this study is based only on a GBM cell line. In this case, we suggest being performed further studies on different cell lines or human isolated glioma cells, and animal models.

In conclusion, it can be concluded that cytotoxicity and apoptosis mechanisms induced by PO are different in various times and concentrations. In the first 24 h and low concentrations, the mechanism is dependent on ROS. Our results suggested that PO could be considered as an inhibitor of NF-κB expression in concentration and time-dependent manner. Nowadays, NF-κB inhibitors are of major importance in cancer and inflammatory disorders in which PO could achieve the goal through different mechanisms.

## Notes

Vafa Baradaran Rahimi, Seyed Hadi Mousavi and Soroush Haghighi contributed equally as first authors.

## Acknowledgements

Authors would like to thank research deputy of Mashhad University of medical sciences regarding financial supports. 

## Conflict of interest

All contributed authors declare no conflict of interest to perform and publishing of this study.

## Figures and Tables

**Table 1:**
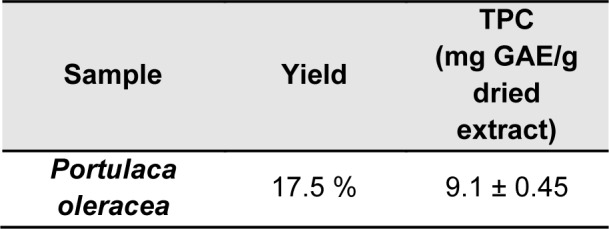
Yield and TPC of *Portulaca oleracea*
